# Severe systemic cytomegalovirus infection in an immunocompetent patient outside the intensive care unit: a case report

**DOI:** 10.1186/s12879-018-3621-8

**Published:** 2019-01-09

**Authors:** Giovanni Carpani, Sergio Foresti, Raffaella Dell’Oro, Guido Grassi, Michele Bombelli

**Affiliations:** 1 Department of Medicine and Surgery, Clinica Medica, University of Milano-Bicocca, San Gerardo Hospital, Via G.B. Pergolesi 33, 20900 Monza, Italy; 2 Division of Infectious Diseases, San Gerardo Hospital, Via G.B. Pergolesi 33, 20900 Monza, Italy

**Keywords:** Cytomegalovirus, Immunocompetent, Colitis, Pneumonia, Valganciclovir

## Abstract

**Background:**

Cytomegalovirus is responsible for an opportunistic infection that can be life threatening in immunocompromised patients, while it is usually mild or completely asymptomatic in immunocompetent subjects. In the recent years, however, some cases of severe cytomegalovirus infection in immunocompetent patients have been reported, showing this to be a less rare occurrence than previously reported.

**Case presentation:**

We report the case of an 83-year-old man, admitted to our hospital for gastroenteritis, complicated by dehydration and severe prothrombin time prolongation due to oral anticoagulant therapy accumulation, who developed hospital-acquired pneumonia; neither of these illnesses responded to several lines of antibiotic therapy. All microbiologic tests were negative, except cytomegalovirus DNA test in blood, which showed high viral load. Antiviral therapy with ganciclovir was then started and a quick favourable response followed. A state of immunodeficiency was excluded, based on normal CD4 count and patient’s clinical history.

**Conclusion:**

Different risk factors for severe cytomegalovirus disease in immunocompetent patients may exist, besides the ones already known, which could be responsible for severe cytomegalovirus disease in immunocompetent patients; thus, these patients should be tested for cytomegalovirus infection, if the clinical picture is compatible, to avoid delay in diagnosis and allow prompt start of specific therapy.

## Background

Cytomegalovirus (CMV) is responsible for a very common infection, which, in the immunocompetent subjects, is often asymptomatic or can present with mild nonspecific symptoms or as a mononucleosis-like syndrome. It is regularly a self-limiting disease, after which the virus remains latent in many different cell types. In the immunocompromised patients, instead, the infection can reactivate and cause a wide range of manifestations, potentially involving many organs and systems, and can often be fatal [1]. During the last years, however, some cases of severe CMV infection in immunocompetent subjects have been reported [2, 3]. Severe CMV infection in non-immunocompromised patients was so shown to be an occurrence not as rare as previously thought, and possibly linked to different risk factors from the ones usually considered.

Here we describe the case of a severe CMV infection in an immunocompetent patient.

## Case presentation

An 83-year-old man was referred to our hospital for the occasional finding, with routine blood tests for oral anticoagulant therapy monitoring, of severe increase of the prothrombin time (PT). The patient also complained weeklong history of fever (up to 39 °C), diarrhoea and vomiting. His medical history included chronic ischemic heart disease, valvular heart disease (mitral valve replacement in 2001 with mechanical prosthesis), permanent atrial fibrillation, arterial hypertension, chronic cerebrovascular disease, peripheral atherosclerosis of the lower extremities, stage 0 chronic obstructive pulmonary disease, stage IIIb chronic kidney disease, chronic gastritis, gastric resection for perforated gastric ulcer in 1975, cholecystectomy for cholelithiasis in 2009, benign prostatic hyperplasia, recent bacterial pneumonia (four months earlier). The patient was admitted to the Department of Internal Medicine.

At presentation the patient was febrile (37.5 °C); physical examination revealed abdominal distension, diffused tenderness and bloating. No stool passage had been observed since the previous day. Blood tests confirmed severe prolongation of PT (22.48, with normal liver function tests), and showed an increase of C reactive protein (CRP, 12.54 mg/dl), normal leucocyte and neutrophil count with mild lymphopenia (860/mm^3^) and monocytosis (1040/mm^3^), acute kidney failure with creatinine 1.7 mg/dl, no acidosis nor increase in lactic acid. Chest X-ray showed mild pulmonary congestion. Abdominal X-ray showed small intestine loop dilation and air-fluid levels, suggesting intestinal obstruction; abdominal computed tomography confirmed these findings and showed wall thickening of the terminal ileum, which the radiologist described as compatible with either inflammatory bowel disease or intestinal ischemia. No surgical indication was established by the general surgeon.

We deemed inflammatory bowel disease unlikely because of the advanced age and the acute onset; intestinal ischemia was also considered improbable because of the intestinal region involved (terminal ileus, with no apparent involvement of the caecum and ascending colon) and because of normal lactate levels. An acute intestinal infection was considered the most likely diagnosis. Samples for blood cultures, stool cultures, stool ova and parasite test and Clostridium difficile toxin assay were collected. Empirical antibiotic therapy with metronidazole and piperacillin-tazobactam was started at admission and vitamin K was administered.

We observed an initial favourable response to therapy: the patient became afebrile, CRP levels decreased (from 12.54 mg/dl to 5.11 mg/dl); constipation resolved, with appearance of loose stool, that rapidly progressed to overt diarrhoea. Microbiologic tests all came back negative. This positive trend was however transient, and on the fifth day since admission, the patient developed fever (38.2 °C) and a drop in peripheral oxygen saturation was observed. Samples for blood and urine cultures were collected and came back negative; chest X-ray showed a basal opacity of the right lung. Levofloxacin was then added to ongoing therapy. No response to therapy was observed and during the next three weeks the patient was persistently febrile, respiratory function progressively worsened and bilateral opacities appeared at the chest X-ray controls; furthermore, the stool remained loose or liquid. Several microbiologic tests were performed and they all were negative: two more blood culture sets (obtained during temperature spikes), two more stool cultures, stool ova and parasite test and Clostridium difficile toxin assay, urine culture, serology for Chlamydia, Legionella and Mycoplasma, Legionella and Pneumococcal urinary antigen, serum galactomannan assay, stool cultures for Mycobacterium tuberculosis and Quantiferon. Echocardiography was negative for vegetations. Colonoscopy was also performed to investigate persistent diarrhoea and showed a single linear erosion in front of the ileocecal valve, compatible with infectious colitis, with no abnormal findings in the terminal ileum; two biopsies of the caecum were performed.

Antibiotic therapy was repeatedly modified: initial therapy with piperacillin-tazobactam, metronidazole and levofloxacin was replaced with cefepime and linezolid, then with meropenem, linezolid and azithromycin, lastly with meropenem and ceftaroline. None of these changes resulted in clinical improvement.

At the same time, the patient’s general conditions worsened: heart failure progressed with development of anasarca, respiratory function was impaired so that respiratory support by non invasive continuous positive airway pressure was needed. Furthermore, consciousness was progressively compromised, with the onset of delirium.

After almost three weeks of persistent fever, samples were then collected for Adenovirus and Rotavirus stool test, assays for detection of influenza from nasal and throat swabs, Brucella and Leishmania serology, CMV DNA detection in blood. CMV DNA test was positive, with 57,679 copies/ml, while all other tests were negative. The patient was then assessed for risk factors for immunodeficiency: HIV serology was negative, CD4 count was normal (925/mm^3^) and the patient had never been on immunosuppressive drugs and had no history of organ transplantations or malignancy. CMV serology showed low immunoglobulin M (IgM) titre and high immunoglobulin G (IgG) titre, suggesting reactivation of CMV infection. To assess the patient for further organ involvement, ophthalmologic evaluation was performed and no ocular manifestations of CMV infection were detected. Histological examination of colon biopsies showed mild non-specific chronic inflammation, but no CMV-induced cytopathic alterations; immunohistochemical study was negative for CMV antigens and PCR on biopsy specimens was negative for CMV.

Antibiotic therapy was then stopped and the patient was started on antiviral therapy with ganciclovir. Quick defervescence followed, the patient remained stably afebrile and consciousness steadily improved. Over the next three weeks, we observed a progressive recovery in respiratory function, with almost complete resolution of lung opacities, and a decrease in watery diarrhoea, although it did not resolve completely during hospital stay. CMV DNA was no longer detected in blood after 14 days since the start of antiviral therapy. The patient was kept on intravenous therapy with ganciclovir for a month and was then discharged from hospital in stable and globally improved clinical conditions, with the indication to continue antiviral therapy with oral valganciclovir, which was prosecuted for at least three and a half months until admission to the Neurology Department for a serious ischemic stroke. The patient was then lost to follow up. Our patient experienced mild elevation of alkaline phosphatase (up to 199 U/l) and gamma-glutamyl transferase (up to 83 U/l) as the only side effects of ganciclovir, without indication to interruption of antiviral therapy. A spontaneous decrease of both was later observed, with a progressive return to normal levels.

## Discussion and conclusions

We presented here a case of presumptive severe CMV disease in a seemingly immunocompetent patient, who recovered only with the use of antiviral therapy. In the recent years, some cases of severe CMV disease in immunocompetent patients have been reported; however, it is still a rare event, even though more common than it was considered in the past. In our opinion, our patient’s clinical picture might entirely be explained by multi-organ involvement by CMV disease, with affection of bowel (terminal ileum and colon) and lungs. Both sites are possible targets of CMV infection, even in immunocompetent subjects; colon, in particular, appears to be the organ most commonly involved, while CMV pneumonia has been reported as a rarer occurrence [2].

In our patient, high viral load in blood was detected, but we could not find any direct signs of end organ involvement. Microscopic examination of colon biopsies did not reveal typical cytopathic alterations of CMV infection. However, this might be due to insufficient sampling of colonic mucosa (only two samples were drawn, considering the mild endoscopic manifestations of the inflammatory process). As for pneumonia, the patient was not clinically stable enough to undergo bronchoscopy and we could not isolate CMV from bronchoalveolar lavage fluid. This procedure might have sped up the diagnostic process, possibly allowing us to avoid further attempts at antibiotic therapy, which were later proven to be unnecessary, and to start antiviral therapy earlier. Likewise, CMV PCR on sputum was not performed, as the patient was not able to expectorate at the time when CMV DNA was detected in blood. Since end organ involvement by CMV infection could not be confirmed, it might be argued that, in our patient, viremia might have been a consequence of reactivation of CMV infection in a critically ill subject, a well known and widely described condition, in which the usefulness of antiviral treatment has not been established yet [4]. Nonetheless, the steady recovery of our patient’s general conditions, followed by a marked improvement of respiratory function and decrease of diarrhoea, suggests CMV infection as the primary cause of the described clinical picture, together with compatible symptoms and signs [2, 5, 6]. Furthermore, all microbiologic tests were negative, except CMV DNA in blood.

Only a few elements have been suggested as possible risk factors for severe CMV disease in immunocompetent patients, and only steroid use and recent blood transfusions were reported as independent risk factors for development of CMV colitis in the immunocompetent host [7]. Many cases of severe CMV disease have been reported in immunocompetent critically ill patients [4]; however, even in subjects admitted in the Intensive Care Units (ICU), few risk factors have been reported, the only ones described being sepsis, blood transfusions, corticosteroid use, acute respiratory distress syndrome [8] and degree of inflammation [9]. In our patient clinical manifestations of CMV infection appeared at home, aggravated during hospitalization in the Internal Medicine Department and the whole course of the disease was spent outside the ICU.

Considering the increase in reports of severe CMV disease in immunocompetent subjects, it is likely that further risk factors exist, even in non-ICU patients, which can trigger or facilitate CMV reactivation and have yet to be identified. Our patient suffered from stage IIIb chronic kidney disease, which might be a possible and so far unrecognized risk factor for reactivation of CMV infection. Chronic kidney disease is associated with a reduction in immune function, and this may be the case, even in the presence of a normal CD4 count. It is known that chronic kidney disease can cause retention of metabolites that can exert an inhibitory effect on activation of immune system cells; furthermore, it can impair the barrier function of intestinal mucosa and reduce the number of circulating lymphoid cells [10]. However, with the exception of an increased risk of tubercolosis in patients with end-stage renal failure [11], in our knowledge, renal insufficiency has not yet been described as a risk factor for a specific bacterial or viral infection. Moreover, our patient’s CD4 count was normal, which reflects a preserved cell-mediated immunity, normally involved in the protection against CMV infection reactivation, regardless of kidney function. It appears indeed unusual that, in the presence of a normal CD4 count, the infection could reactivate and, possibly, cause severe disease with end organ involvement.

In conclusion, we deem that CMV infection should also be suspected in non-immunosuppressed patients, when the clinical picture is compatible with or suggestive of this disease, even in a non-ICU population. Taking this possibility into account early during the diagnostic process could lead to a timely diagnosis and start of specific antiviral therapy, which might in turn change the course of this possibly fatal disease.

## Abbreviations

CMV: cytomegalovirus
CRP: C reactive protein
ICU: intensive care unit
IgG: immunoglobulin G
IgM: immunoglobulin M
PT: prothrombin time
